# Contribution of extracellular vesicles in normal hematopoiesis and hematological malignancies

**DOI:** 10.1016/j.heliyon.2021.e06030

**Published:** 2021-01-20

**Authors:** Soudeh Ghafouri-Fard, Vahid Niazi, Mohammad Taheri

**Affiliations:** a Urogenital Stem Cell Research Center, Shahid Beheshti University of Medical Sciences, Tehran, Iran; b Department of Tissue Engineering and Applied Cell Sciences, School of Advanced Technologies in Medicine, Shahid Beheshti University of Medical Sciences, Tehran, Iran; c Urology and Nephrology Research Center, Shahid Beheshti University of Medical Sciences, Tehran, Iran

**Keywords:** Extracellular vesicle, Hematopoiesis, Hematological malignancy

## Abstract

Extracellular vesicles (EVs) are lipid bilayer-enclosed microparticles that have prominent roles in the intercellular crosstalk. EVs are secreted after fusion of endosomes with the plasma membrane (exosomes) or shed from the plasma membrane (microvesicles). These microparticles modulate bone marrow microenvironment and alter differentiation and expansion of normal hematopoietic cells. EVs originated from mesenchymal stromal cells have been shown to enhance expansion of myeloid-biased hematopoietic progenitor cells. In addition, megakaryocytic microparticles stimulate differentiation of hematopoietic stem and progenitor cells into mature megakaryocytes. The ability of EVs in induction of maturation and expansion of certain hematopoietic cells has implications in transfusion medicine and in targeted therapeutic modalities. Important prerequisites for these interventions are identification the specific targets of EVs, transferred biomolecules and molecular mechanisms underlying the fate decision in the target cells. EVs are also involved in the pathogenesis and progression of hematological malignancies including acute leukemia and multiples myeloma. In the current review, we provide a summary of studies which evaluated the significance of EVs in normal hematopoiesis and hematological malignancies.

## 1 Introduction

As lipid bilayer-enclosed particles, extracellular vesicles (EVs) have prominent roles in the intercellular communication. Microvesicles (MV) and exosomes include two important kinds of EVs [1]. While MVs have sizes more than 100 nm and are released through direct budding, exosomes have sizes between 30 to 100 nm and are produced via fusion of the multivesicular bodies with the plasma membrane [2]. They can transmit several biomolecules such as proteins, lipids and nucleic acids between cells, thus affecting numerous physiological and pathological features in both receiver and donor cells [3]. The transferred molecules can change the phenotype of recipient cells by changing gene expression [4]. In the context of cancer, EVs change stroma properties and tumor angiogenesis by affecting gene expression in target cells, therefore enhancing cancer progression and metastasis [5]. Such method of cell communication has been detected among both prokaryotes and eukaryotes [3]. Moreover, EVs have been shown to be derived from most kinds of cells and have been detected in almost all types of biofluids [6, 7]. In addition to their systemic effects, EVs are involved in the determination of the fate of hematopoietic stem cells (HSCs) and progenitor cells through modulating the bone marrow microenvironment [8]. Besides, the transmission of biomolecules via EVs contributes in the pathogenic processes during development and progression of hematological malignancies. The International Society for Extracellular Vesicles has suggested Minimal Information for Studies of Extracellular Vesicles guidelines for different aspects of researches in this field such as accrediting a certain role to EVs or classification of different types of EVs. These guidelines have emphasized on the importance of providing specific data further than simple explanation of function of EVs including contaminations in the process of EVs preparation [9]. In the current review, we provide a summary of studies which evaluated the significance of EVs in normal hematopoiesis and hematological malignancies. For this purpose, we searched PubMed and google scholar databases using the key words “extracellular vesicles” or “exosome” and “hematopoiesis” or “leukemia” or “multiple myeloma” or “myeloma”. Papers which provided the origin of EVs, their target cells, transported molecules and functional effect of EVs were included in the study. Those with inadequate data were excluded.

## 2 EVs in normal hematopoiesis

Normal hematopoiesis is influenced by biomolecules that exist in EVs. For instance, EVs originated from mesenchymal stromal cells have been shown to enhance expansion of myeloid-biased hematopoietic progenitor cells. This speculation is based on an experiment in murine stromal cells where EVs derived from these cells stimulated loss of quiescence in hematopoietic stem and progenitor cells (HSPCs) and enhanced expansion of myeloid progenitors. Such effects were exerted through the MyD88 adapter protein. Notably, inhibition of TLR4 could suppress this process to some extent. Canonical NF-κB pathway and subsequent induction of Hif-1α and CCL2 were also involved in this process [10]. Megakaryocytic microparticles have also been demonstrated to stimulate differentiation of HSPCs into mature megakaryocytes. Being distinctive from exosomes, these microparticles specifically target HSPCs without significant impacts on other cells such as mesenchymal stem cells (MSCs). They also specifically expand megakaryocytic cells. These microparticles deliver their ingredients through endocytosis as well as direct fusion with HSPCs membrane. HSPCs receive these microparticles through macropinocytosis and lipid raft-mediated routes. Such interactions between these two kinds of cells are mediated through binding of microparticles with different molecules such CD54 (ICAM-1), CD11b, CD18 and CD43 on the surface of HSPCs [11]. In addition, EVs mediate the impact of bone marrow MSCs on the HSC. The RNA component of these EVs has prominent role in the determination of fate of CD34 + cell obtained from umbilical cord blood. These EVs significantly alter expression profile of these CD34 + cells. Notably, MPL, ANXA1, EGR2 have been among differentially expressed genes. These EVs also altered viability and differentiation properties of CD34+. Moreover, they enhanced expression of several genes particularly genes coding for chemokines, cytokines and their receptors, thus affecting chemotaxis of various bone marrow cells and hematopoietic reconstruction [12]. The miR-126-enclosing microvesicles which are induced by granulocyte colony-stimulating factor have functional roles in the bone marrow microenvironment. Being incorporated by HSPCs, stromal cells and endothelial cells, these microvesicles suppress expression of VCAM-1 on these cells, thus influencing the regulation of HSPCs transport between the bone marrow and peripheral sites [13, 14]. The observed alterations in EVs and their enclosed miRNAs during the lifetime have provided clues for participation of these particles in age-related stem cell dysfunction. An animal study has demonstrated remarkable difference in miRNA signature of bone marrow EVs between the young and aged mice showing over-expression of the miR-183 cluster in EVs isolated from elder animals. Functional studies showed the impact of aging and oxidative stress on miRNA load of EVs in the bone marrow milieu and their possible contribution in stem cell senescence and osteogenic differentiation [15]. Membrane-derived vesicles produced by embryonic stem cells have stem cell-specific components that facilitate self-renewal and proliferation of adult stem cells. These vesicles have been shown to promote survival and proliferation of murine hematopoietic progenitor cells (HPCs), increase expression of early pluripotent early HSC markers in HPCs, and stimulated phosphorylation of a number of proteins in MAPK/AKT pathway. Further studies revealed that embryonic stem cells-derived particles can enhance expansion of HPC by inducing them with a number of ligands such as Wnt-3 and increasing their pluripotency [16]. Table 1 summarizes the results of studies which evaluated the contribution of EVs in normal hematopoiesis.

**Table 1:** Extracellular vesicles in normal hematopoiesis.

EVs origin	Type of EVs	Target cell	Transported molecules	Functional effect of EVs	Reference
Reticulocytes	EVs	Macrophage	Transferrin receptor (TR)	Recycling of TR in the maturation of erythropoiesis in bone marrow	[17]
MSCs	Exosomes	HSC/HPC	Unidentified	Activation of TLR4 signaling and myeloid bias proliferation	[10]
Megakaryocytes (Mk)	Microparticles	HSC/HPC	Mk-RNA	Activation of ICAM-1, CD63, CD18 and CD11b and differentiation of HSC/HPC into Megakaryocytes	[11]
BM-MSCs	Exosomes	HSC/HPC	Unidentified	Reduction of caspase 3/7 activity, increasing engraftment of CD34 + umbilical cord blood cells, up-regulation of chemotactic factors	[12]
Stromal cell (SC) stimulated by G-CSF	Exosomes	Stromal cell, endothelial cell, HSC/HPC	miR-126	Downregulation of VCAM-1 and mobilization of HSC/HPC into the peripheral bone marrow	[13]
Adipose-derived MSCs and BM-MSCs	Heterogeneous population of EVs	HSC/HPC	TLR-4	Differentiation of myeloid linage and decreasing stemness of HSC/HPC	[18]
Aged mouse BM cell	Exosomes	Young mouse BM stromal cell	miR-183-5p	Decreasing proliferation of SC and osteogenic differentiation	[15]
Mouse ESC	Microvesicles enriched in exosomes	HSC/HPC	Wnt3, Oct4	Upregulation of SCL, HoxB4, GATA2, and phosphorylation of MAPK p24/44 resulted in the expansion of HSC/HPC	[16]
HSC/HPC	Exosomes	HSC/HPC	TPO, ANGPTL2, ANGPTL3	Autocrine signaling and maintaining cell stemness	[19]

EVs: extracellular vesicle, TR: transferrin receptor, MSC: mesenchymal stem cell, HSC: hematopoietic stem cell, HPC: Hematopoietic progenitor cell, TLR4: Toll-Like Receptor 4, Mk: megakaryocyte, ICAM: Intercellular Adhesion Molecule 1.

## 3 Role of EVs in hematological malignancies

EVs also mediate different roles in hematological malignancies.

### 3.1 EVs in AML

Both primary acute myelocytic leukemia (AML) and AML cell lines have been shown to produce exosomes that are transferred to bystander cells. These EVs contain numerous coding and noncoding RNAs which are involved in the pathobiology of AML. EVs entrance into bone marrow stromal cells results in the modulation of their potential in growth factors production. The signals transmitted by these EVs change the proliferative, angiogenic, and migratory features of cocultured stromal and hematopoietic progenitor cells [20]. EVs also transfer endoplasmic reticulum stress from the AML xenograft to bone marrow stroma, leading to over-expression of core unfolded protein response element and osteogenic differentiation of mesenchymal stem cells [21]. Exosomes containing miR-486 have been shown to modulate hypoxia-associated erythroid differentiation of erythroleukemia cells via regulating expression of Sirt1 [22]. This miRNA has been recognized as a hypoxia-induced transcript in erythroleukemia cells which promotes the growth and erythroid differentiation of these cells [22]. Notably, the protein and RNA components of exosomes produced by stromal cells in patients with AML have been shown to be altered. These stromal cells produce exosomes containing TGFB1, miR-155 and miR-375. These biomolecules might also modulate response of AML cells to therapeutic modalities. For instance, exosomal miRNAs might suppress expression of pro-apoptotic genes or genes involved in the process of cell differentiation, thus liberating the AML cell from dependence to kinase proteins [23]. An important study in human subjects havee shown over-expression of protein and TGF-β1 levels in AML patients at the time of diagnosis compared with control exosomes. Notably, these parameters were reduced after induction chemotherapy, enhanced during consolidation chemotherapy and returned to normal levels in long term remission. Treatment of natural killer cells with AML exosomes containing TGF-β1 forms led to a significant decrease in NKG2D expression [24]. The AML-derived EVs also generate a self-protecting leukemic microenvironment that enhances proliferation and survival of AML cells, while inhibiting normal hematopoiesis. These effects are induced by down-regulation of HSC-supporting proteins such as CXCL12, KITL and IGF1 in bone marrow stromal cells [25]. In agreement with this study, EVs that are originated from AML cells have been shown to inhibit hematopoiesis through transferring miRNAs targeting c-MYB [26].

### 3.2 EVs in chronic leukemia

Exosomes originated from patients with chronic myelogenous leukemia (CML) patients are enriched in amphiregulin (AREG), therefor inducing EGFR signaling in stromal cells. Activation of this signaling pathway leads to over-expression of SNAIL, MMP9 and IL-8. Prior exposure of stromal cells with CML cells exosomes has enhanced expression of annexin A2 which increases the adhesion of leukemic cells to the stromal monolayer, facilitating the growth and invasion of leukemic cells [27]. CML-derived exosomes have also been demonstrated to induce IL-8 production in bone marrow stromal cells, modulating leukemia cell malignant features [28]. Bcr/Abl hybrid gene can also been transported by EVs to normal neutrophils, resulting in the expression of its fusion product in a subset of cells [29]. Microvesicles which are present in the plasma samples of patients with B-cell chronic lymphocytic leukemia (CLL) change during the course of disorder. These microvesicles can induce the AKT/mTOR/p70S6K/HIF-1α axis in CLL-bone marrow stem cells leading to alterations in the β-catenin pathway and up-regulation of cyclin D1 and c-myc in these cells [30].

### 3.3 EVs in myelodysplastic syndrome and multiple myeloma

Microvesicles released from MSCs from patients with myelodysplastic syndrome (MDS) have been shown to alter CD34 + cells characteristics [31].

Multiple myeloma (MM) MSCs have also been shown to produce EVs that are conveyed to MM cells, thus changing tumor growth. Exosomal levels of miRNA such as miR-15a were different between MM and normal MSCs. Besides, EVs originated from MM bone marrow MSCs had greater amounts of oncogenes, cytokines, and adhesion molecules. While these EVs enhanced MM tumor growth, normal bone marrow MSC-derived EVs suppressed the growth of MM cells [32]. MM patients have been shown to have higher amounts of EVs which have CD9 and CD38 markers. EVs of MM patients have procoagulant functions evident by augmented thrombin production and both tissue factor and procoagulant phospholipids activity. Procoagulant function of EVs has been decreased during treatment particularly following induction therapy with bortezomib, cyclophosphamide, and dexamethasone [33]. In MM-derived exosomes, fibronectin has been identified as an important heparan sulfate-binding ligand and facilitator of exosome-cell interactions. Heparan sulfate not only captures fibronectin, but also it functions as a receptor for fibronectin in target cells [34]. Experiments in animal models of MM have shown that MM-derived exosomes transfer several angiogenesis-associated molecules which increase angiogenesis and enhance endothelial cell growth. STAT3, c-Jun N-terminal kinase, and p53 have been identified as targets of MM-derived exosomes in endothelial and BM stromal cells. These exosomes have been shown to increase the growth of myeloid-derived suppressor cells in naive animals via induction of the STAT3 pathway. Besides, MM-derived exosomes have increased levels of inducible nitric oxide synthase and promoted the immunosuppressive effects of BM MDSCs in animal models [35].

Table 2 summarizes the results of studies which evaluated the participation of EVs in the pathogenesis or progression of hematological malignancies.

**Table 2:** Extracellular vesicles in malignant hematological disorders.

Condition	EVs origin	Type of EVs	Target cell	Transported molecules	Functional effect of EVs	Reference
AML-M6	Malignant blasts	EVs	HSC/HPC	miR-486-5p	In hypoxia situation increase erythroid differentiation	[22, 36]
AML	AML-MSCs	Exosomes	HSC/HPC	Unidentified	Increase chemo-resistance to Cytarabine and Quizartinib	[23]
AML	Blast cell	Exosome	stromal cell	IGF-IR mRNA	Upregulate VEGF expression	[20]
AML	Blast cell	Exosome	stromal cell	BMP-2 protein	Induction of osteogenic differentiation and unfolded protein response	[21]
AML	Blast cell	Exosome	stromal cell	TGF-b1	Damage NK-cell activity by decreasing NKG2D expression	[24]
AML	Blast cell	EVs	Stromal cell	Enrichment of EGF	Migration and proliferation of tumor cell	[23]
AML	AML cell lines	Exosomes	Leukemic cell	Unidentified	leukemic cell survival, increase expression of DKK1, reduce normal hematopoiesis by diminishing of hematopoietic stem cell supporting factors such as CXCL12, KITL, and IGF1	[25]
AML	Malignant cell	EVs	HSC/HPC	miR-155, miR-150, and miR-375	downregulation of c-MYB, SCF, CXCR4, c-kit	[26]
AML	Blast cell	EVs	Immune Cells	TGF-β1, MICA/MICB, FasL	proliferation and differentiation of Tregs, Inhibition of NKG2D on NK	[24, 37, 38, 39]
AML	Blast cell	EVs	Stromal cell	Unidentified	Downregulation of CXCL12, SCF, IGF-1, and suppression of hematopoiesis, downregulation of DKK1	[25]
AML	Blast cell	EVs	HSC/HPC	miR-150/155	Downregulation of cMYB and CXCL12 that result in decrease clonogenicity and induce mobilization of HSC/HPC to peripheral blood	[26, 40, 41, 42]
AML and MDS cells	Blast cell	EVs	MSCs	miR-7977	Downregulation of PCBP1, jagged-1, SCF, ANGPT1 (reduce HSC supportive factors)	[43]
MDS	MSCs	EVs	CD34 + cells	miR-10a/15a	transcriptional dysregulation of P53 and MDM2 and change the viability and clonogenicity of HSC/HPC	[31]
MDS	MDS-MSCs	MVs	HSCs CD34+	Unidentified	Downregulation of MDM2 in HSCs CD34+	[31]
MM	MM-MSCs/human stromal cell line HS-5/healthy donor MSCs (HD-MSCs)	Exosomes	MM cell	Unidentified	HD-MM exosomes reduce cell proliferation but MM-MSCs exosomes increase cell proliferation and adhesion capacity resulting in tumor growth.	[32]
MM	MM-MSCs/HD-MSCs	Exosomes	MM cell	Unidentified	Drug resistance to Bortezumib, increase in BCL-2 and inhibition of apoptosis, altered phosphorylation of p38, p53	[44]
MM	MM-MSCs	MVs	MM cell	Unidentified	Increased viability, proliferation, and migration capacity, and enhanced phosphorylation of MAPKs	[45]
MM	HD-MSCs	Exosomes transfected with miR340 and miR365 mimics	MM cell	miR340	Suppress cMET translation	[46]
MM	5TGM1 cells And C57BL6/KalwRij mouse model	EVs	MM cell	Unidentified	Increase osteoclast activity and inhibit osteoblast differentiation and sensitize the MM cells to Bortezomid	[47]
MM	MM-MSCs	Exosomes	MSCs	Unidentified	Increase the secretion of IL-6, enhance expression of APE1 and NF-kB, inhibit the osteoblastic differentiation of BM-MCs	[48]
CML	Malignant cell	EVs	HUVEC	miR-17–92 cluster, enriched miR-92a	EC migration and tube formation	[49]
CML	Malignant cell	EVs	HUVEC	miR-126	tumor growth and progression	[50]
CML	Malignant cell	EVs	HUVEC	Unidentified	Overexpression of IL-8, increase in ICAM-1 and VCAM-1, induction of angiogenesis	[51]
CML	Malignant cell	EVs	Stromal cell	Amphiregulin (AREG)	Enhancement of proliferation of tumor cell and activation of EGFR	[27]
CML	Blast cell	EVs	Stromal cell	Amphiregulin (EGFR-ligand)	Upregulation of IL8 and MMP9, change the structure of BM niche and promote the proliferation of CML cell	[27]
DLBCL	Malignant cell	EVs	Stromal cell, autocrine	Wnt3a, Wnt proteins	Wnt signaling pathway, metastasis	[52]
CLL	Malignant cell	EVs	Stromal cell	miR-202-3p, miR-628-3p, and miR-1290	Proliferation of tumor cell, diminish the anti-tumor effect	[53]
B-cell CLL	Malignant cell	EVs	Stromal cell	Phospho-receptor tyrosine kinase, Axl	Survival of CLL B-cells, secretion of VEGF	[30]
ATLL	Malignant cell	EVs	MSCs	miR-21, miR-155, and VEGF	Migration and induction of angiogenesis in MSCs	[54]
ALL	Malignant cell	EVs	Target ALL Cells	Galectin-3	Activation of the NFkB pathway and drug resistance	[55]

AML: acute myeloid leukemia, SC: stromal cell, G-CSF: Granulocyte colony-stimulating factor, VCAM: vascular cell adhesion molecule, BM: bone marrow, ESC: embryonic stem cell, MAPK: mitogen-activated protein kinase, TPO: thrombopoietin, ANGPTL: Angiopoietin-like proteins, BMP: bone morphogenic protein, IGF-IR: Insulin-like growth factor 1 receptor, TGF-b1: Transforming growth factor-beta, MM: multiple myeloma, HD: healthy donor, CXCL12: C-X-C motif chemokine 12, SCF: Stem cell factor, MDS: Myelodysplastic syndromes, EGFR: epithelial growth factor receptor, CML:chronic myeloid leukemia, IL8: interleukin 8, MMP9: matrix metalloprotease 9, EGF: Epidermal growth factor, DLBCL: Diffuse Large B-cell Lymphoma, CLL: Chronic Lymphocytic Leukemia, CML: Chronic Myelogenous leukemia, ATLL: Adult T-cell Leukemia/Lymphoma, HUVEC: Human Umbilical Vein Endothelial Cells, EC: Endothelial cell.

## 4 Malignant cells-derived EVs as diagnostic and prognostic markers

The presence of EVs in the body fluids including saliva, urine, peripheral blood and other body fluids indicates the potential of these vesicles in diagnostic and prognostic procedures [56]. Notably, peripheral blood has been considered as a massive source of these vesicles based on the high concentration of EVs in the serum, constitutive release of these vesicles into the circulation and the association between release speed and cell activity or pathologic conditions [56]. EVs comprise a surplus of clinically pertinent molecules namely proteins and RNA molecules derived from the original cell. Thus, RNA and protein signature of these vesicles reflects the specific abnormality of cells particularly the presence of malignant transformation which is accompanied by extensive changes in the transcriptome/proteome. The prognostic role of EVs in human cancers is highlighted by several investigations that reported significant increase in the amount of measurable EVs in the blood samples in patients affected with malignancies compared with healthy controls [57, 58, 59]. Besides, distribution of dimensions of vesicles, their phenotype, or ingredients vary during the course of disease [59, 60] indicating diagnostic and prognostic impacts of these vesicles. Notably, many RNA profiling strategies in cancer detection rely on the amount and pattern of EV-enclosed RNAs as the existing RNA in serum and plasma is shielded from the highly active RNases when being kept inside the EVs [4, 61]. Therefore, EVs are considered as perfect targets for liquid biopsy [62]. One of the pioneer studies in the hematological malignancies has identified the presence of high amounts of noncoding Y RNA hY4 in EVs in the plasma samples of CLL patients indicating a diagnostic role for this molecule [63]. Moreover, this RNA molecule has been shown to stimulate immunosuppressive features in monocytes through enhancement of PD-L1 expression [63], possibly affecting the CLL prognosis.

## 5 Discussion

EVs have been shown to mediate critical functions during normal hematopoiesis and in the course of initiation and progression of hematological malignancies. The cell-cell communication mediated by EVs has important effects in bone marrow milieu and regulation of innate immune responses [10]. The ability of EVs in induction of maturation and expansion of certain hematopoietic cells has implications in transfusion medicine and in targeted therapeutic modalities. EVs can be used to enhance differentiation of a certain lineage of hematopoietic cells, blood component manufacturing or modulating immune response of the recipient. Important prerequisites for these interventions are identification the specific targets of EVs, transferred biomolecules and molecular mechanisms underlying the fate decision in the target cells. The EV-mediated crosstalk between bone marrow MSCs and HSCs has also practical significance in cord blood transplantation. Moreover, as these EVs alter expression of mobility factors in the recipient cells, they provide novel modalities for selective mobilization of HSPCs. RNA-containing exosomes also participate in the pathogenesis of hematological malignancies. For instance, miRNA-486 not only controls normal erythropoiesis but also it promotes growth of CML progenitors and their response tyrosine kinase inhibitors [36] and growth and differentiation of erythroleukemia cells [22]. Therefore, a certain component within EVs might affect phenotype of different cells.

Finally, EVs which are released into bone marrow microenvironment might affect response of malignant cells to therapeutic modalities including tyrosine kinase inhibitors. Therefore, EVs components not only predict patients' outcome and their response to therapy, but also they represent potential manipulable resources for conferring drug resistance. Alteration in exosomal level of certain proteins might also reveal responses to chemotherapy. Moreover, the exosomal content may reflect the existence of residual disease in patients thought to be in complete remission [24]. Malignant cells-derived EVs are also potential diagnostic and prognostic markers as they can be detected in the biofluids. Assessment of the levels of transferred molecules in EVs is an applicable method for patients' follow-up after implementation of a therapeutic option. The content of EVs has implications in the design of anti-cancer treatment modalities as thermal- and oxidative stresses have been shown to increase production of NKG2D ligand-containing EVs with immunosuppressive effects in leukemia and lymphoma cells [38].

Taken together, EVs contain several protein and RNA molecules that could alter lineage differentiation, expansion and function of bone marrow cells in both physiological and pathologic conditions. The ability of these microparticles in specific targeting of the recipient cells potentiates them as vehicles for combating drug resistance in hematological malignancies. Moreover, malignant cells-derived EVs are potential diagnostic and prognostic markers in this kind of malignancy.

## Declarations

### Author Contribution statement

All authors listed have significantly contributed to the development and the writing of this article.

### Funding statement

This research did not receive any specific grant from funding agencies in the public, commercial, or not-for-profit sectors.

### Data availability statement

Data will be made available on request.

### Declaration of interests statement

The authors declare no conflict of interest.

### Additional information

No additional information is available for this paper.
